# Response to Comments on “High Altitude Pulmonary Edema in an Experienced Mountaineer. Possible Genetic Predisposition”

**DOI:** 10.5811/westjem.2015.7.28039

**Published:** 2015-10-20

**Authors:** Kenneth S. Whitlow

**Affiliations:** Kaweah Delta Medical Center, Department of Emergency Medicine, Visalia, California

In reply:

We appreciate the letter to the editor and are pleased to respond regarding our recent case study regarding high altitude pulmonary edema in an experienced mountaineer. The letter raises some valid questions regarding our treatment decisions. With this, as with most emergency department (ED) patients, it must be understood that the initial treatment reflected the breadth of our differential diagnosis. The patient was receiving nebulizers as he was wheeled into the department for evaluation for a possible asthmatic condition. An initial chest x-ray in the ED revealed “multiple nodular opacities” and our bedside read could not exclude bilateral pulmonary edema of unknown etiology. Although retrospectively the patient’s history is consistent with High-altitude pulmonary edema (HAPE), for a patient with continued low oxygen saturation despite supplemental oxygen, diffusely coarse breath sounds, and a broad differential it seemed appropriate for a trial of Furosemide for hypervolemic causes of his apparent pulmonary overload as he was not hypotensive. Regarding his continued treatment outside of our department, we are unable to comment specifically given that we were his emergency providers, but again, continuing Dexamethasone for the possibility that he may have been experiencing some degree of reactive airway or an asthmatic response seems relatively low risk with significant potential gains. Given the patient’s rapid response to treatment during his very brief stay his differential was readily narrowed and he was discharged home in excellent condition after a very short stay. This case report was focused on the possible familial or genetic predisposition to HAPE and not intended as a complete review of the prevention and treatment of altitude related illnesses. Perhaps this was a limitation of our manuscript. We are pleased that our case report was read with such interest and welcome further discussion at any time.

